# The TFRC as a prognostic biomarker and potential therapeutic target in cervical cancer: a preliminary study

**DOI:** 10.3389/fonc.2025.1523137

**Published:** 2025-04-15

**Authors:** Jing Wang, Wen An, Ziyao Pang, Manyin Zhao, Anli Xu, Junwei Zhao

**Affiliations:** ^1^ Department of Gynaecology, Yantai Yuhuangding Hospital Affiliated to Medical College of Qingdao University, Yantai, Shandong, China; ^2^ Department of Pathology, Tonglu First People’s Hospital, Hangzhou, China

**Keywords:** cervical cancer, cervical intraepithelial neoplasia, TFRC, bioinformatics, immune cell infiltration

## Abstract

**Background:**

Early detection and treatment of CIN or early-stage cervical cancer lead to better clinical outcomes compared to treating advanced-stage patients. Thus, specific biomarkers for the diagnosis and prognosis of CIN and early-stage cervical cancer should be urgently explored.

**Methods:**

We analyzed tumor based on genes closely related to OS in the database with GSE63514, GSE7803, GSE9750 and TCGA data sets, the top 20 core genes were screened out. Notably, transferrin receptor (TFRC) emerged as a prioritized candidate due to its dual role in cellular iron homeostasis and oncogenic signaling. However, the exact role of TFRC in the development and progression of cervical cancer remains unclear. We then used various bioinformatics methods and mathematical models to analyze those data, aiming to investigate the clinical significance of TFRC in cervical cancer and illustrate its association with tumor immunity. In addition, the molecular function and mechanisms of TFRC were revealed by gene ontology, Kyoto Encyclopedia of Genes and Genomes, and gene set enrichment analysis. Immunohistochemistry was employed to assess TFRC protein expression in 19 cervical cancers, 16 HSILs and 15 normal cervical tissues.

**Results:**

TFRC was highly expressed in CESC in the TCGA and GSE9750 datasets. Meanwhile, the expression of TFRC was correlated with pathological stage, lymph node metastasis, malignant degree of cervical lesions and HPV infection status. Our analysis confirmed that TFRC expression was higher in CESC tissues compared to normal cervical tissues, and it was also elevated in HSIL relative to normal tissues, as determined by IHC staining. Increased TFRC expression was linked to decreased overall survival (OS) (*p* = 0.024), disease-specific survival (DSS) (*p* = 0.009), and progression-free interval (PFI) (*p* = 0.007) in CESC patients. In different clinical stages, pathological T stages, and pathological N stages, higher TFRC expression was significantly associated with worse survival for OS and DSS. We constructed a nomogram model, TFRC contributed significantly to the prognosis and exhibited good predictive power for the OS and the DSS. Finally, we confirmed that immunosuppression in cervical cancer is closely related to high TFRC expression.

**Conclusions:**

TFRC exhibits significant diagnostic and prognostic value in cervical cancer.

## 1 Background

Cervical cancer ranks as the fourth leading cause of cancer-related deaths among women globally, with over 604,127 new cases and 341,831 deaths reported in 2020 (1). Persistent infection with high-risk human papillomavirus (HR-HPV) is the main pathogenic factor for cervical cancer and intraepithelial neoplasia (CIN), HR-HPVs have been detected in 99.7% of cervical cancers (2). Although preventive HPV vaccination and screening programs have been implemented, the global burden of cervical cancer remains significant, particularly in regions with limited access to these measures (3). HPV-infected cells decrease interferon secretion, disrupting a crucial mechanism for antiviral immune stimulation (4). Although most HPV infections are cleared or become latent, those that persist can progress to CIN (5). CIN 1 (low-grade, mild dysplasia) is a benign cervical dysplasia state associated with viral replication, and conservative treatment is recommended. Because it is expected that 70% -90% of CIN1 lesions will subside within 2-3 years (6). Untreated CIN3 (high-grade, severe dysplasia) can develop into invasive cervical cancer, and less than 20% of which have been proven to regress (7). Timely detection and treatment of CIN or early-stage cervical cancer yield better clinical outcomes than treating advanced-stage patients. However, early diagnostic biomarkers are currently lacking, and most cancer cases are detected in the later stages (8, 9). In addition, standard treatments for cancer include surgery, radiation therapy, and platinum-based chemotherapy (10). Recent statistical data shows that late stage and recurrent cases have poor response to these interventions, with a five-year survival rate of approximately 17% for metastatic diseases (11). The introduction of targeted therapies and immune checkpoint inhibitors provides new avenues for improving the prognosis of these late-stage patients (12). Therefore, specific biomarkers for the diagnosis and prognosis of CIN and early cervical cancer urgently need to be explored.

This study aims to analyze tumors by examining genes that are closely associated with overall survival (OS) using data from the GSE63514, GSE7803, GSE9750, and TCGA-CESC databases. The top 20 core genes were screened out, these are KIFC1, KIF14, HELLS, TK1, GINS2, WDR76, PCNA, DSG2, MCM5, SYNGR3, APOBEC3B, CHAF1B, TMPO, NUP62CL, RIBC2, PLA2G7, ARHGAP4, TFRC, GAD1, SPP1. Notably, transferrin receptor (TFRC) emerged as a prioritized candidate due to its dual role in cellular iron homeostasis and oncogenic signaling. However, the underlying mechanism of TFRC in the occurrence and malignant progression of cervical cancer is still unclear. Among these, we ultimately selected the TFRC gene as the primary focus of our research.

The TFRC gene encodes two distinct types of transferrin receptors, namely TFR1 and TFR2, which serve as the most crucial receptor-mediated regulatory factors for cellular iron uptake, as highlighted in the findings of Qin et al. (13). Iron is an important micronutrient and is central to various biological processes including oxygen transport, DNA replication, and redox reactions (14). The intricate interactions between iron metabolism, ferroptosis, and tumorigenesis have recently attracted great attention in cancer biology (15). It is worth noting that ferroptosis is an iron dependent programmed cell death (PCD) characterized by the accumulation of reactive oxygen species (ROS). This accumulation can be offset by iron chelators and lipophilic antioxidants (16). Several studies have documented that the TFRC gene is abnormally overexpressed in various human tumors, including but not limited to liver cancer, glioblastoma, and colorectal cancer. The expression levels of TFRC are significantly elevated in human tissues, particularly in metastatic tumors, and this overexpression is directly correlated with poor prognosis (17, 18; W. 19, 20). However, the exact role of TFRC in the development and progression of cervical cancer remains unclear.

In light of these findings, this study harnessed extensive sample data from various databases, complemented by biochemical experiments, to thoroughly investigate the expression levels and potential clinical applications of TFRC in the context of cancer. Additionally, we employed advanced bioinformatics techniques to delve into the underlying mechanisms of TFRC and its significant role in immunotherapy, ultimately providing valuable recommendations for enhancing cancer treatment strategies.

## 2 Materials and methods

### 2.1 Data collection and analysis

We obtained TFRC-related expression data and clinical information from the TCGA Pan-Cancer Database. TCGA tumor abbreviations are listed in **Supplementary Table S1**. We used TCGA to obtain tumor tissue and normal tissue to analyze TFRC expression. Cervical cancer microarray data were obtained from the GEO database, including GSE63514 (platform: GPL570), GSE7803(platform: GPL96), GSE9750(platform: GPL96) and GSE7410 (platform: GPL1708).

### 2.2 Correlation and enrichment analyses

We calculated the Pearson correlation coefficient using TCGA data to assess the correlation between TFRC and other mRNAs in cervical squamous cell carcinoma and endocervical adenocarcinoma (CESC). This analysis aimed to identify the top 300 genes correlated with TFRC expression for further enrichment analysis. Gene ontology (GO) analysis was performed using the EnrichGO function of the Cluster Profiler R package. Kyoto Encyclopedia of Genes and Genomes (KEGG) analysis was performed by enrichment of KEGG functions by cluster contour R packets. Gene set enrichment analysis (GSEA) is performed using the gseGO, gseKEGG, and gse path functions of the Cluster Profiler R package.

### 2.3 TideSCORE analysis of TFRC expression in CESC

We collected initial data and relevant clinical features from RNA sequencing available in the TCGA dataset, which was accessed following established guidelines and policies. The TIDE algorithm plays an important role in predicting potential Immune checkpoint blockade (ICB) responses.

### 2.4 Immune cell infiltration

The infiltration scores of TCGA pan-cancer data estimated using CIBERSORT were downloaded. Next, we categorized TCGA samples into high and low TFRC expression groups using the median expression level to compare immune cell infiltration.

### 2.5 Establishment and evaluation of the nomogram models

In the present study, univariate Cox regression analysis for overall survival (OS) was performed in tumors where TFRC can affect prognosis, including overall survival (OS), and disease-specific survival (DSS), tumors with p <0.05. We constructed separate nomogram models, which provide effective and convenient methods for predicting overall survival (OS) and disease-specific survival (DSS) in individual patients. The calibration curves were performed to assess the prediction accuracy of the nomograms at 1-year, 3-year, and 5-year.

### 2.6 Prognosis analysis

We employed Kaplan-Meier analysis with a log-rank test to evaluate the relationship between TFRC expression and clinical outcomes, including overall survival (OS), progression-free interval (PFI), and disease-specific survival (DSS) in CESC from TCGA, displaying survival curves with p < 0.05. Besides, the receiver operating characteristic curve (ROC) were drawn in tumors where TFRC can affect prognosis.

### 2.7 Correlation analysis between TFRC expression and clinical features

We investigated how TFRC expression correlates with key clinical parameters, including gender, T stage, N stage, and pathologic stage, in cancers where TFRC impacts prognosis.

### 2.8 Immunohistochemistry

After surgery, we collected paraffin-embedded samples, which included 19 cervical cancers, 16 high-grade squamous intraepithelial lesions (HSILs), and 15 normal cervical tissues. These samples were deparaffinized and rehydrated following standard protocols for immunohistochemical (IHC) examination. The primary antibodies and antigen retrieval regimes used were as follows: anti-TFRC (Affinity [AF5343]). We calculated the positive area (%) using ImageJ and performed statistical analyses with GraphPad Prism version 7.0.1. Statistical significance was evaluated using two-tailed t-tests: *p < 0.05, **p < 0.01, and ***p < 0.001.

### 2.9 Statistical analysis

We compared the differences between the two groups using the Wilcoxon rank-sum test and assessed their correlation with the Spearman rank test. Univariate and multivariate Cox proportional hazard regression were performed to screen the factors influenced the prognosis. Kaplan-Meier analysis with log-rank test was used to survival analysis. We conducted statistical analyses using R (version 4.0.2), considering p-values of less than 0.05 as statistically significant, with thresholds set at *p < 0.05, **p < 0.01, ***p < 0.001, ****p < 0.0001.

## 3 Results

### 3.1 Pan-cancer TFRC expression analysis

**Figure 1:**
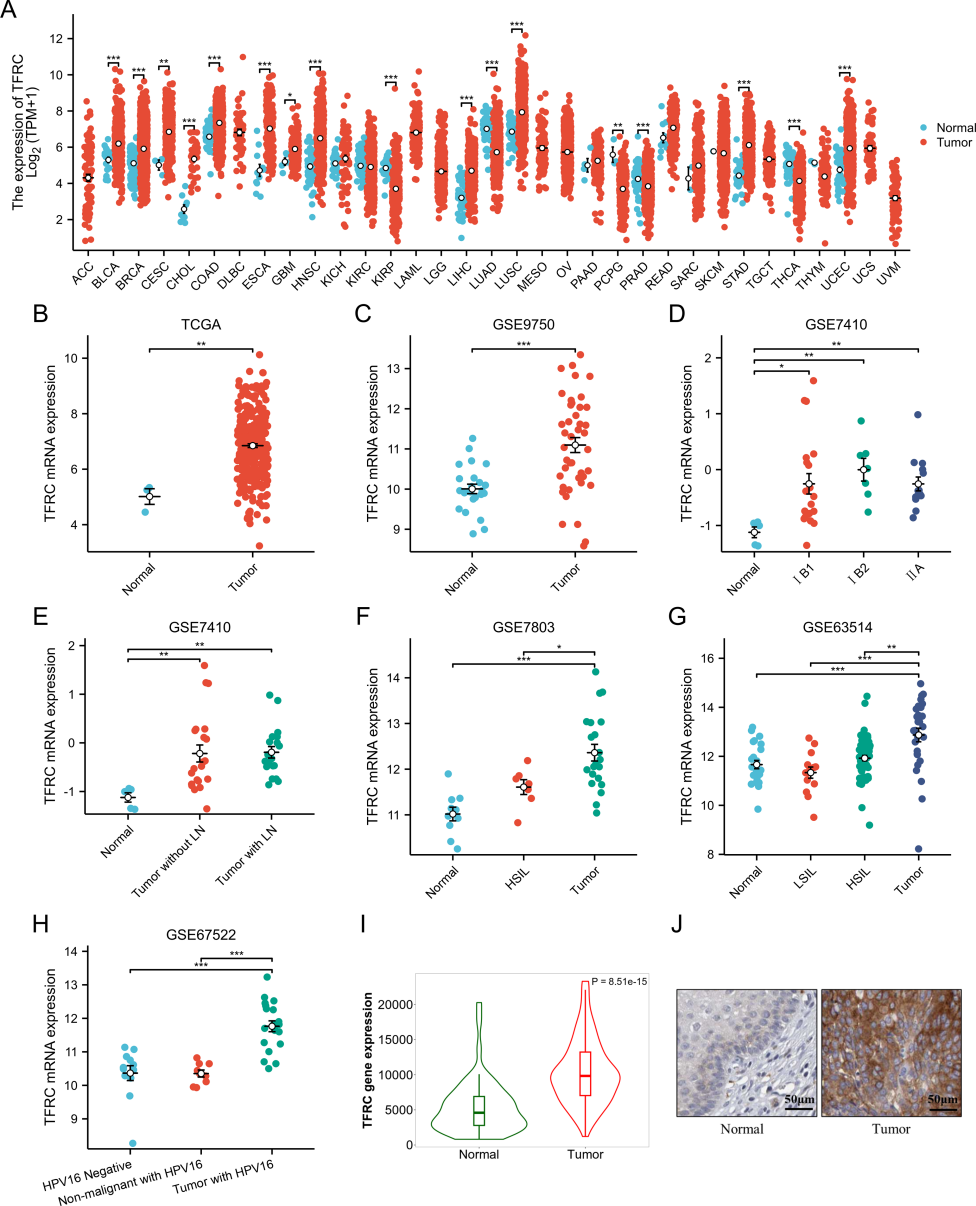
Pan-cancer TFRC expression analysis. **(A)** The mRNA expression of TFRC in 33 tumors in TCGA_GTEx samples. **(B)** TFRC expression in tumor and normal tissues in cervical cancer from TCGA data. **(C)** TFRC expression in normal cervical surface epithelium and cervical cancer epithelial component from GSE9750. **(D)** TFRC expression in normal cervical surface epithelium and cervical cancer epithelial component (IB1, IB2 and IIA) from GSE7410. **(E)** TFRC expression in normal cervical surface epithelium and cervical cancer epithelial component (with/without LN) from GSE7410. **(F)** TFRC expression in normal cervical surface epithelium, high-grade squamous intraepithelial lesions (HSIL) and cervical cancer epithelial component from GSE7803. **(G)** TFRC expression in normal cervical surface epithelium, low-grade squamous intraepithelial lesions (LSIL), high-grade squamous intraepithelial lesions (HSIL) and cervical cancer epithelial component from GSE63514. **(H)** TFRC expression in normal cervical surface epithelium (HPV negative), non-malignant with HPV16 and cervical cancer epithelial component (with HPV16) from GSE67522. **(I)** TFRC expression in tumor and normal tissues in cervical cancer from HPA data. **(J)** The IHC images of TFRC in normal and tumor tissues extracted from the HPA. *p < 0.05, **p < 0.01, and ***p < 0.001.

To clarify TFRC expression across various cancers, we analyzed 33 tumor types from the TCGA pan-cancer dataset. Our results indicated that TFRC expression was elevated in 12 tumor types compared to their respective normal tissues, including bladder (BLCA), breast (BRCA), cervical (CESC), cholangiocarcinoma (CHOL), colon (COAD), esophageal (ESCA), glioblastoma (GBM), head and neck squamous cell carcinoma (HNSC), liver (LIHC), lung (LUSC), stomach (STAD), and uterine (UCEC) cancers. In contrast, it was expressed lower in KIRP, LUAD, PCPG, PRAD and THCA than corresponding normal tissues (**Figure 1A**). We also found that TFRC expression was significantly elevated in cervical cancer (CESC) according to both TCGA and GSE9750 datasets (**Figures 1B, C**). Furthermore, we examined the relationship between TFRC expression and clinicopathological features in CESC. The results indicated a positive correlation between TFRC expression and factors such as pathological stage, lymph node metastasis, and the malignancy level of cervical lesions (**Figures 1D–G**). Notably, TFRC expression in cervical cancer tissues was significantly greater than in high-grade squamous intraepithelial lesions (HSIL) (**Figures 1F, G**). Meanwhile, TFRC expression was correlated with HPV infection status (**Figure 1H**), the expression of TFRC in tumor with HPV16 was significantly higher than that in non-malignant with HPV16 (p < 0.05). Further, we studied the protein expression of TFRC was significantly higher in the cervical cancer tissues than in normal tissues on the HPA (**Figure 1I**, p < 0.0001), and representative immunohistochemical (IHC) images of normal and tumor tissues of cervix were extracted (**Figure 1J**).

### 3.2 Relationship between TFRC expression and prognosis of cancer patients

**Figure 2:**
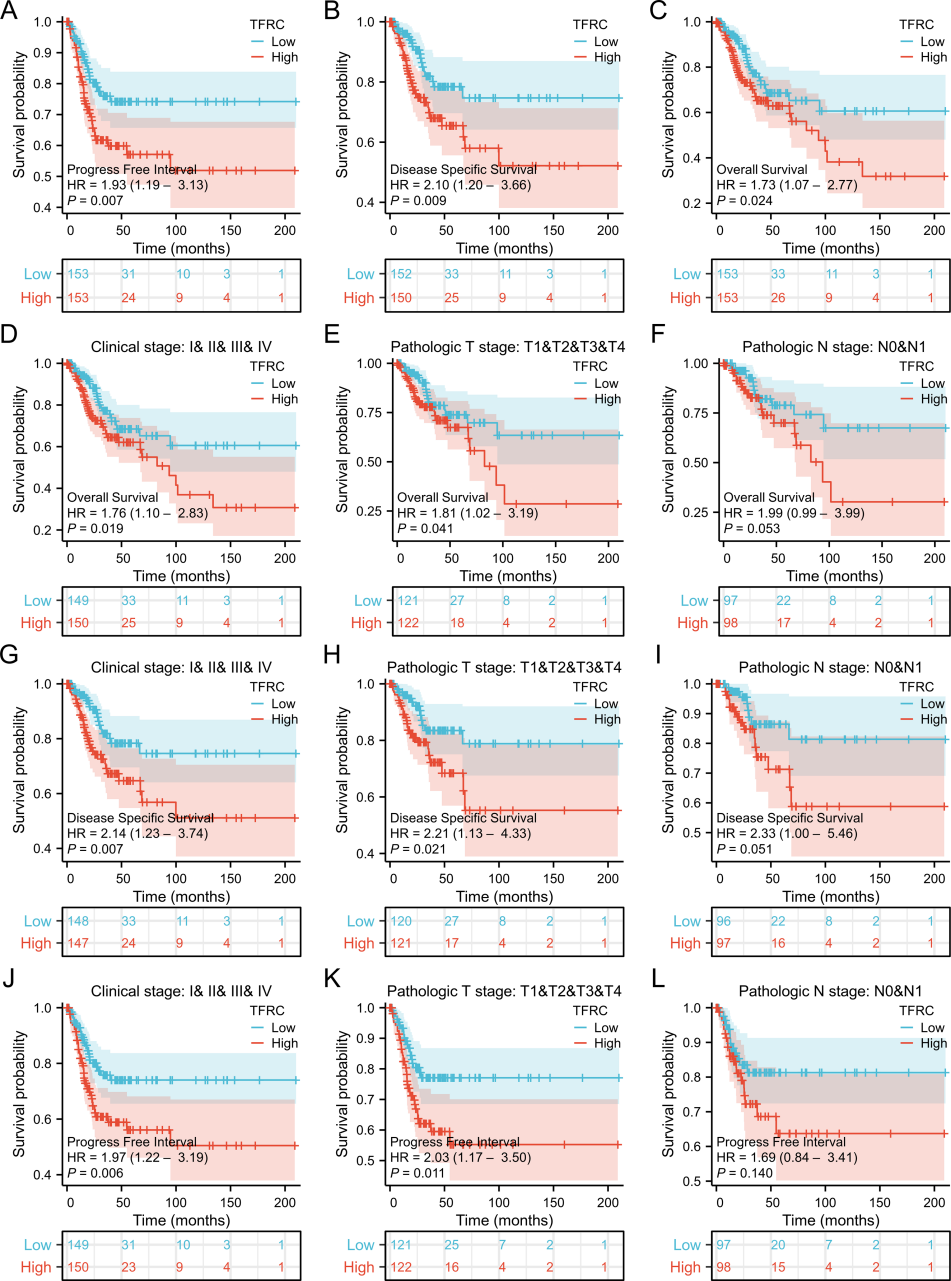
Correlation between TFRC expression and cancer prognosis. **(A–C)** Kaplan–Meier analysis of overall survival(HR=1.73, p = 0.024), disease specific survival(HR=2.10, p = 0.009) and progress free interval(HR=1.93, p = 0.007) in TCGA. **(D-F)** Kaplan–Meier analysis of clinical stage(HR=1.76, p = 0.019), pathological T stage (HR=1.81, p = 0.041), and pathological N stage(p = 0.053) in CESC for OS. **(G-I)** Kaplan–Meier analysis of clinical stage(HR=2.14, p = 0.007), pathological T stage(HR=2.21, p = 0.021), and pathological N stage (p = 0.051) in CESC for DSS. **(J-L)** Kaplan–Meier analysis of clinical stage (HR=1.97, p = 0.006), pathological T stage (HR=2.03, p = 0.011), and pathological N stage (p = 0.140) in CESC for PFI. Results with COX p <0.05 are shown.

To assess the role of TFRC in predicting cancer prognosis, we analyzed the relationships between TFRC expression and OS, DSS, and PFI in the TCGA cohort (**Figure 2**). Higher TFRC expression was associated with reduced OS (HR=1.73, p = 0.024), DSS (HR=2.10, p = 0.009), and PFI (HR=1.93, p = 0.007) in CESC (**Figures 2A–C**). Additionally, we investigated the associations between TFRC expression and clinical stage, pathological T stage, and pathological N stage in CESC. For OS, higher TFRC expression significantly correlated with worse survival in both clinical stage (HR=1.76, p = 0.019) and pathological T stage (HR=1.81, p = 0.041). However, TFRC expression did not show a significant association with OS in pathological N stage (p = 0.053) (**Figures 2D–F**). For DSS, TFRC overexpression reduced survival in clinical stage (HR=2.14, p = 0.007) and pathological T stage (HR=2.21, p = 0.021), whereas TFRC overexpression did not significantly reduce survival in pathological N stage (p = 0.051) (**Figures 2G–I**). For PFI, higher TFRC expression was significantly associated with worse survival in clinical stage (HR=1.97, p = 0.006), pathological T stage (HR=2.03, p = 0.011), whereas TFRC expression was not significantly associated with worse survival in pathological N stage (p = 0.140) (**Figures 2J–L**).

### 3.3 Construction and evaluation of nomogram models in cervical cancer

**Figure 3:**
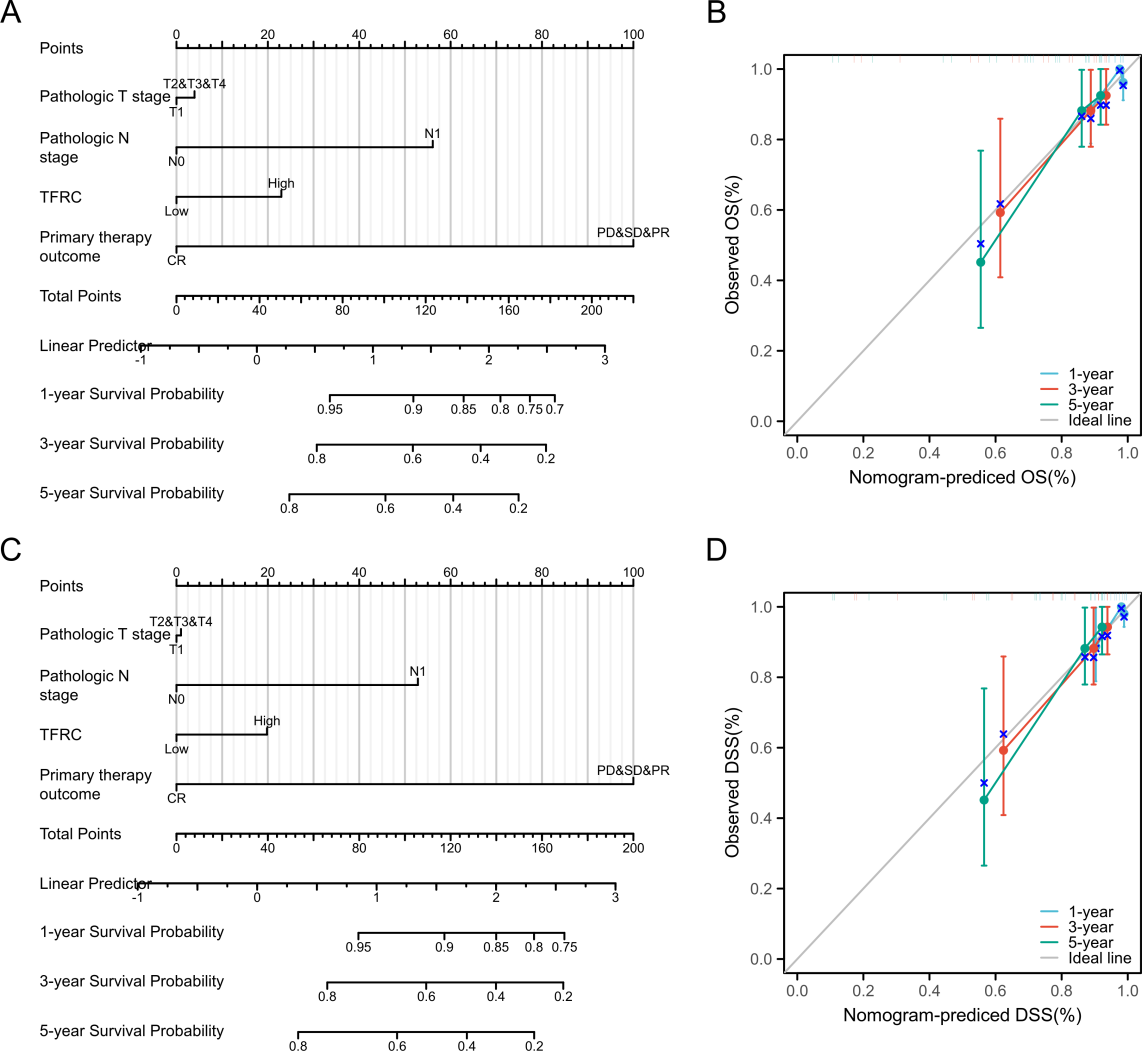
Nomogram models were established and evaluated in cervical cancer. **(A)** Establishment of a nomogram model incorporating TFRC expression for OS. **(B)** Calibration curves were used to evaluate the nomogram model for OS at 1-year, 3-year, and 5-year. **(C)** Building a nomogram model containing TFRC expression for DSS. **(D)** The 1-year, 3-year and 5-year calibration curves were used to evaluate the prediction accuracy of the nomogram model for DSS.

To further investigate the impact of TFRC expression on cancer prognosis, we performed univariate Cox regression analysis on overall survival (OS) and disease-specific survival (DSS). This analysis aims to determine how TFRC influences prognosis (**Tables 1**, **2**). Based on the univariate Cox regression results, we selected samples to create column chart models that validate prognostic values. Calibration curves were then used to assess the predictive accuracy of these models for 1, 3, and 5 years. The results indicated that TFRC significantly contributed to prognosis in the nomogram models, demonstrating strong predictive ability for both overall survival (OS) and disease-specific survival (DSS) (**Figures 3A, C**). The calibrated 1-year, 3-year, and 5-year survival prediction curves indicated that the nomogram model had high accuracy in predicting OS and DSS (**Figures 3B, D**).

**Table 1:** Univariate and multivariate Cox regression analysis for OS in CESC.

Characteristics	Total(N)	Univariate analysis		Multivariate analysis	
		Hazard ratio (95% CI)	*P* value	Hazard ratio (95% CI)	*P* value
TFRC	306				
Low	153	Reference		Reference	
High	153	1.726 (1.074 - 2.773)	**0.024**	1.760 (0.682 - 4.539)	0.242
Clinical stage	299				
Stage I	162	Reference		Reference	
Stage II	69	0.813 (0.413 - 1.600)	0.548	0.502 (0.109 - 2.313)	0.377
Stage III	46	1.390 (0.707 - 2.734)	0.340	0.532 (0.117 - 2.421)	0.414
Stage IV	22	4.376 (2.354 - 8.137)	**< 0.001**	1.985 (0.234 - 16.820)	0.530
Pathologic N stage	195				
N0	134	Reference		Reference	
N1	61	2.844 (1.446 - 5.593)	**0.002**	2.359 (0.922 - 6.035)	0.073
Primary therapy outcome	219				
CR	182	Reference		Reference	
PD&SD&PR	37	13.854 (7.464 - 25.714)	**< 0.001**	6.571 (2.404 - 17.959)	**< 0.001**
Age	306				
<= 50	188	Reference			
> 50	118	1.289 (0.810 - 2.050)	0.284		

CESC, Cervical Squamous Cell Carcinoma and Endocervical Adenocarcinoma; CR, complete response; PD, progressive disease; SD, stable disease; PR, partial response.

Bold values indicate statistically significant differences (p < 0.05).

**Table 2:** Univariate and multivariate Cox regression analysis for DSS in CESC.

Characteristics	Total(N)	Univariate analysis		Multivariate analysis	
		Hazard ratio (95% CI)	P value	Hazard ratio (95% CI)	P value
TFRC	302				
Low	152	Reference		Reference	
High	150	2.101 (1.205 - 3.665)	**0.009**	1.768 (0.644 - 4.852)	0.269
Clinical stage	295				
Stage I	158	Reference		Reference	
Stage II	69	0.895 (0.417 - 1.921)	0.776	0.532 (0.113 - 2.505)	0.425
Stage III	46	1.570 (0.732 - 3.366)	0.247	0.636 (0.137 - 2.961)	0.564
Stage IV	22	5.109 (2.559 - 10.199)	**< 0.001**	2.177 (0.250 - 18.976)	0.481
Pathologic N stage	193				
N0	133	Reference		Reference	
N1	60	3.544 (1.572 - 7.987)	**0.002**	2.272 (0.834 - 6.191)	0.109
Primary therapy outcome	219				
CR	182	Reference		Reference	
PD&SD&PR	37	17.365 (8.883 - 33.948)	**< 0.001**	7.322 (2.608 - 20.553)	**< 0.001**
Age	302				
<= 50	186	Reference			
> 50	116	1.295 (0.761 - 2.204)	0.340		

CESC, Cervical Squamous Cell Carcinoma and Endocervical Adenocarcinoma; CR, complete response; PD, progressive disease; SD, stable disease; PR, partial response; DSS, Disease-specific survival.

Bold values indicate statistically significant differences (p < 0.05).

### 3.4 Correlation and enrichment analyses

**Figure 4:**
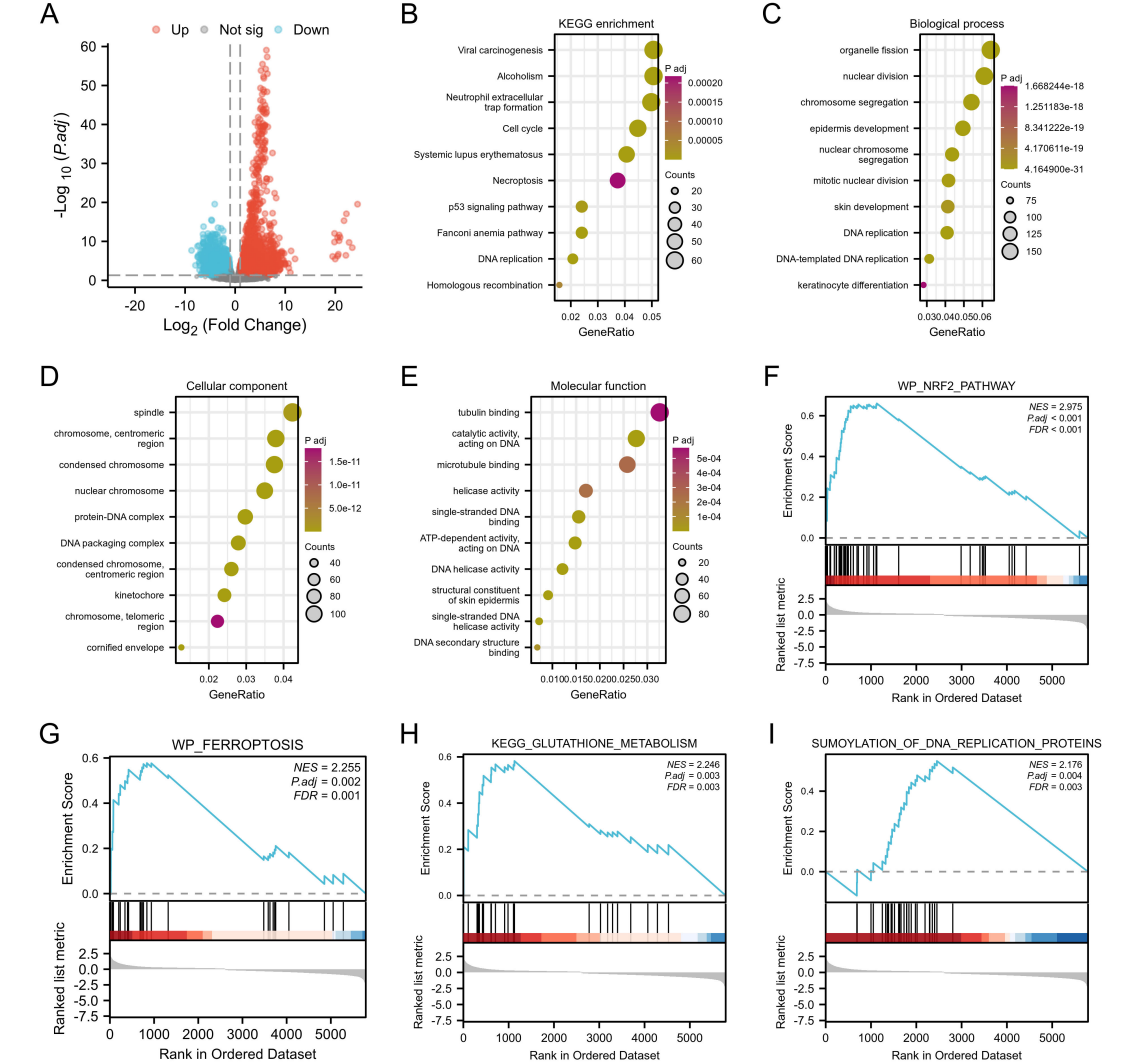
Function and pathway enrichment analyses of TFRC in cervical cancer. **(A)** A volcano plot of the 6,652 differential genes in cervical cancer. **(B)** Significant KEGG pathways of the top 100 genes most positively correlated with TFRC. **(C-E)** Gene Ontology terms of the top 100 genes most positively correlated with TFRC, including biological processes (BP), molecular function (MF), and cell component (CC). **(F-I)** Significant GSEA results of the top 100 genes most positively correlated with TFRC, including KEGG pathways and Reactome pathways.

In this study, a total of 309 CESC related patient data were downloaded, organized, and analyzed from the TCGA database, including 306 tumor samples and 3 normal samples. The DESeq2 R package was used for differential analysis, and a total of 6652 differentially expressed genes were screened, including 4150 up-regulated genes and 2502 down regulated genes. A volcano map was created for 6652 differentially expressed genes (**Figure 4A**). To further elucidate the biological function of TFRC in tumors, we analyzed TCGA data and conducted enrichment analysis on the top 100 genes positively correlated with TFRC. In addition, we also used clustering contour R packages to explore candidate functional pathways associated with the top 100 genes. According to GSEA in KEGG pathways, the TFRC-related pathway mainly focused on “Viral carcinogenesis”, “Alcoholism”, “Neutrophil extracellular trap formation”, “Cell cycle”, and “Systemic lupus erythematosus” (**Figure 4B**). GO analysis suggested that TFRC-related genes may participate in the “organelle fission”, “nuclear division”, “chromosome segregation”, “spindle”, “chromosome, centromeric region”, “condensed chromosome”, “tubulin binding”, catalytic activity, acting on DNA” and “microtubule binding” (**Figures 4C–E**). To further determine the function of TFRC, the GSEA based on the differential expression analysis of TFRC was applied to elucidate the biological function of TFRC. The results suggest that was mainly related to NRF2-pathway, Ferroptosis, Glutathione metabolism and Sumoylation of DNA replication proteins (**Figures 4F–I**).

### 3.5 Correlation between immune cell infiltration and TFRC expression

**Figure 5:**
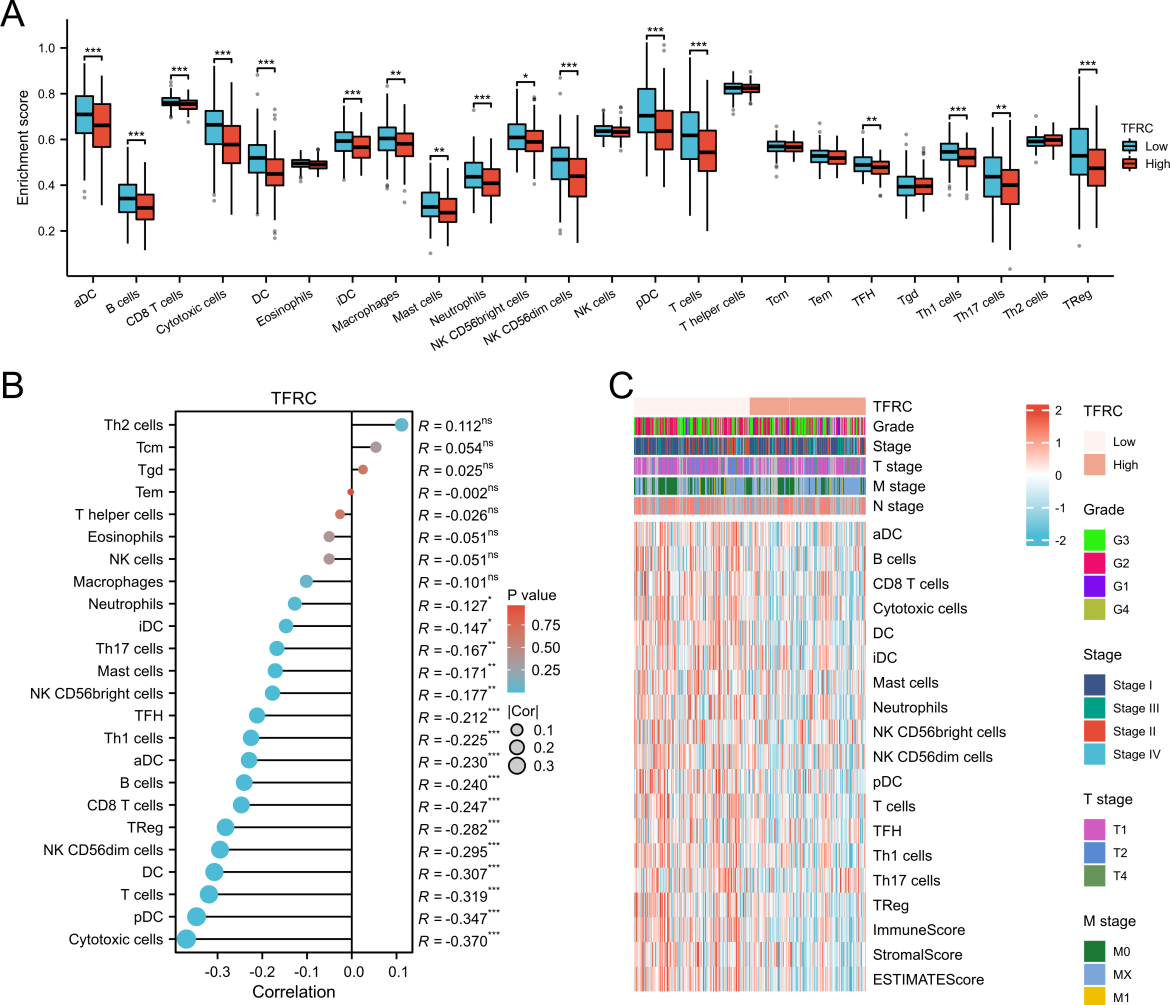
Association between immune cell infiltration and TFRC expression in cervical cancer. **(A, B)** Immune cell infiltration level in the TFRC high expression group and TFRC low expression group in TCGA cohort. **(C)** The abundance of different cell types calculated by MCPCOUNTER was shown in the heatmap. There were significant differences between TFRC expression, tumor stage, grade, and immune cell invasion.

**Figure 6:**
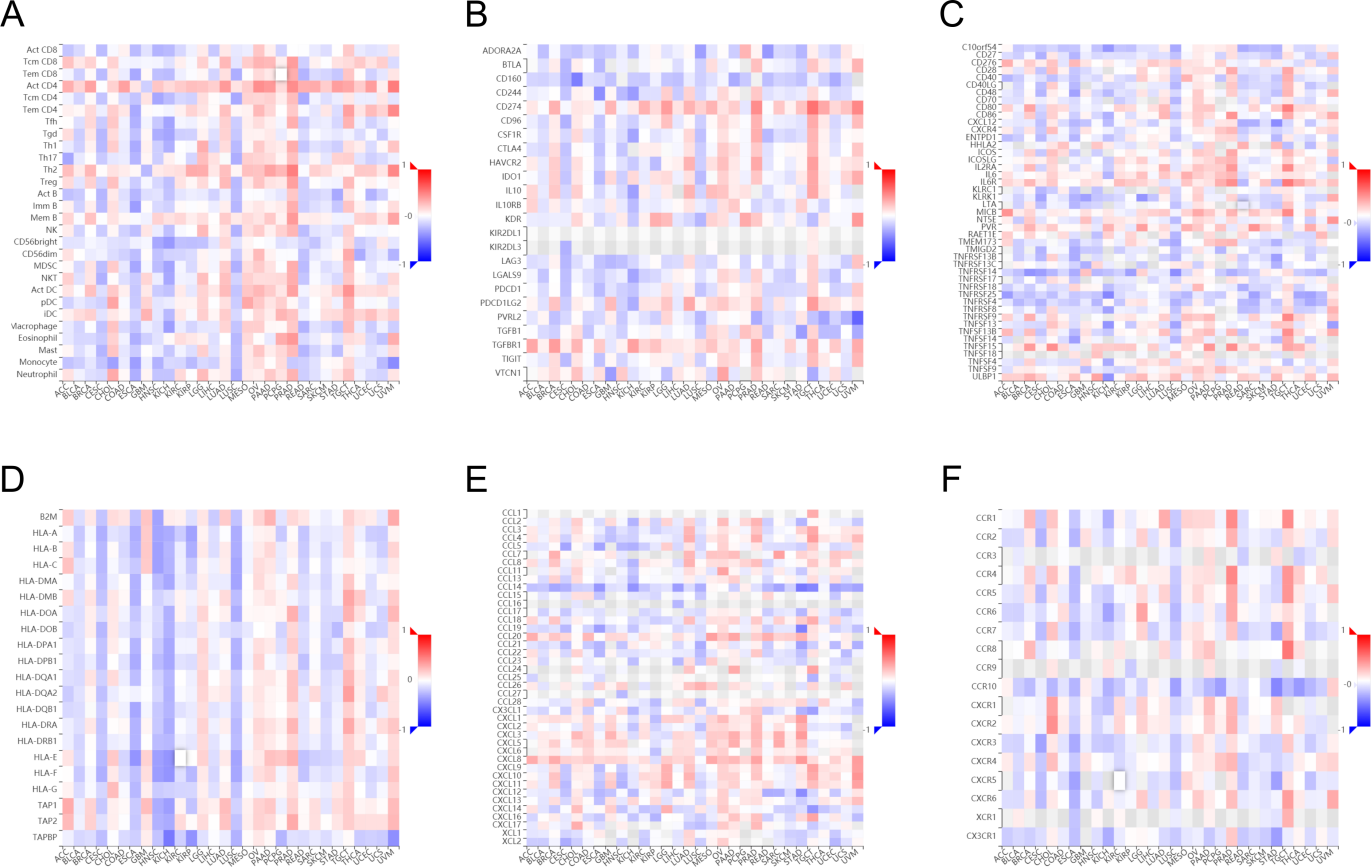
Association between immune cell infiltration and TFRC expression in pan-cancer. Immune cell infiltration level in the TFRC high expression group and TFRC low expression group in TISIDB database **(A)**. Correlations between TFRC and immunoinhibitors **(B)**, immunostimulators **(C)**, MHC molecules **(D)**, chemokines **(E)**, receptors **(F)** are shown in heatmaps, calculated by TISIDB database, where red and blue represent positive and negative correlations, respectively; Color shades represent strong correlations.

We evaluated the infiltration score of immune cells in TCGA of CESC and found that TFRC was correlated with all immune cells (B cells, CD8+ T cells, cytotoxic cells, Th1 cells, macrophages, neutrophils, eosinophils, master cells, dendritic cells, and their subtypes) (**Figure 5**). This suggests that the high expression of TFRC reduces immune cell recruitment, which is closely associated with tumor immunosuppression. Similar results were also obtained by using the TISIDB database to assess the link between immune cell invasion levels and TFRC levels in 30 cancer types (**Figure 6A**). Further analysis of TCGA pan cancer data indicates that TFRC is also associated with immunosuppressive genes. The relationship between TFRC and immunosuppressive genes is significantly negatively correlated in most tumor types, including cervical cancer (**Figures 6B–F**). Based on these findings, we confirmed that immunosuppression in cervical cancer is closely related to high TFRC expression.

**Figure 7:**
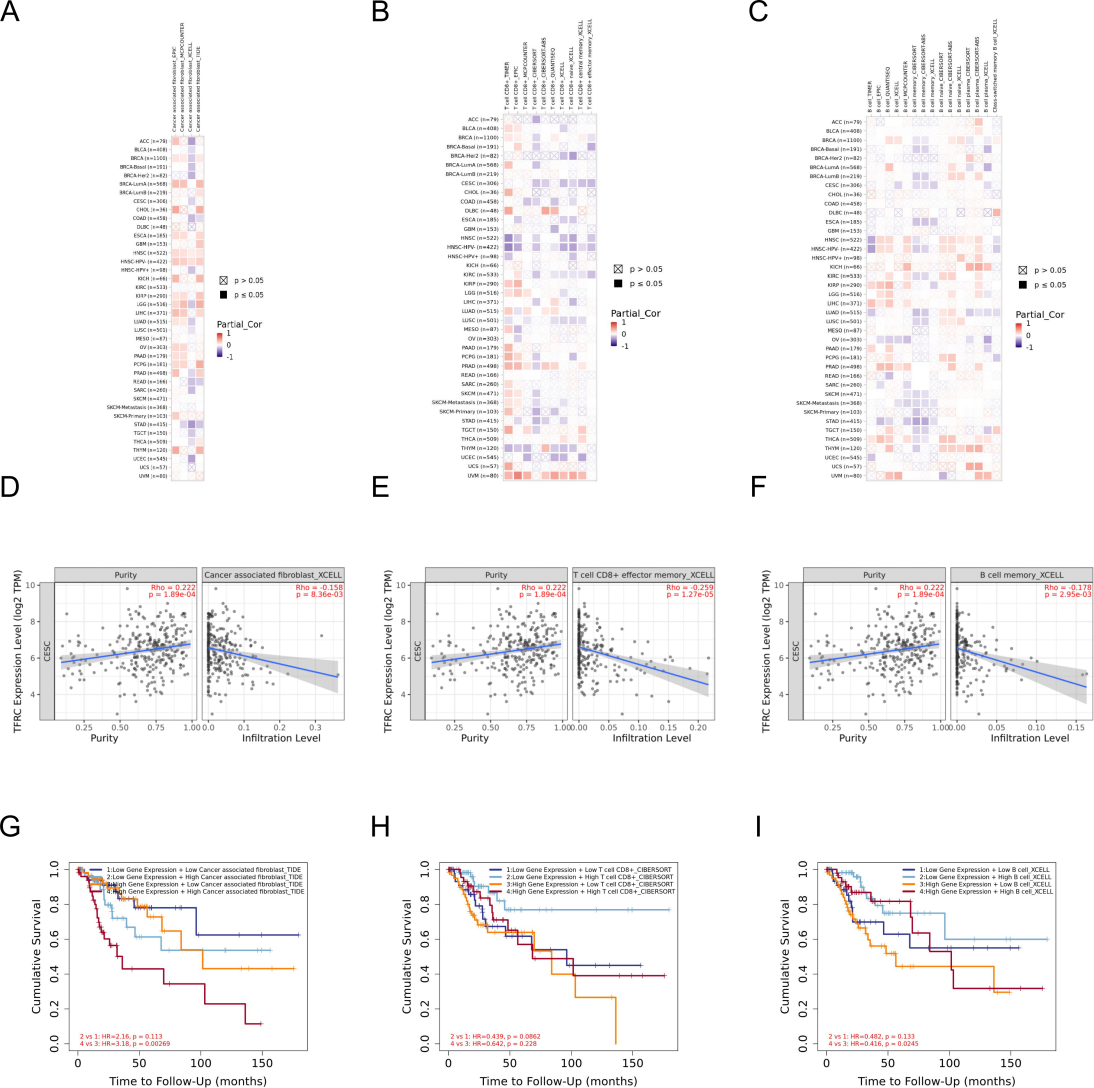
The correlation of TFRC expression and immune cell infiltration. **(A–C)** Heatmaps of correlations between TFRC expression and Cancer associated fibroblast, T cells CD8+, and B cell in TIMER2 database, respectively. **(D-F)** The link between TFRC expression and Cancer associated fibroblast, T cells CD8+, and B cell in XCELL algorithms. **(G-I)** The effect of immune cells infiltration on OS was related to the expression of TFRC.

Immune microenvironment plays a crucial role in the occurrence and development of tumors. To study the relationship between TFRC and immune microenvironment in pan-cancer, the correlation between TFRC expression and immune cells in pan-cancer was carried out by using the GEPIA2 database. The heatmaps of the correlation between TFRC expression and Cancer associated fibroblast (**Figure 7A**), T cells CD8+ (**Figure 7B**), and B cell (**Figure 7C**) were shown. In addition, we used a TIMER2.0 database with XCELL algorithms to further validate our results and found that PDPN expression was negatively correlated with CAFs infiltration levels, T cells CD8+ (**Figure 7B**), and B cell (**Figures 7D–F**). We combined the expression of TFRC and immune cell infiltration to analyze the effect on tumor OS in GEPIA2 that those cell infiltration has an impact on the prognosis in cervical cancer (**Figures 7G–I**).

### 3.6 Experimental verification of the expression of TFRC

**Figure 8:**
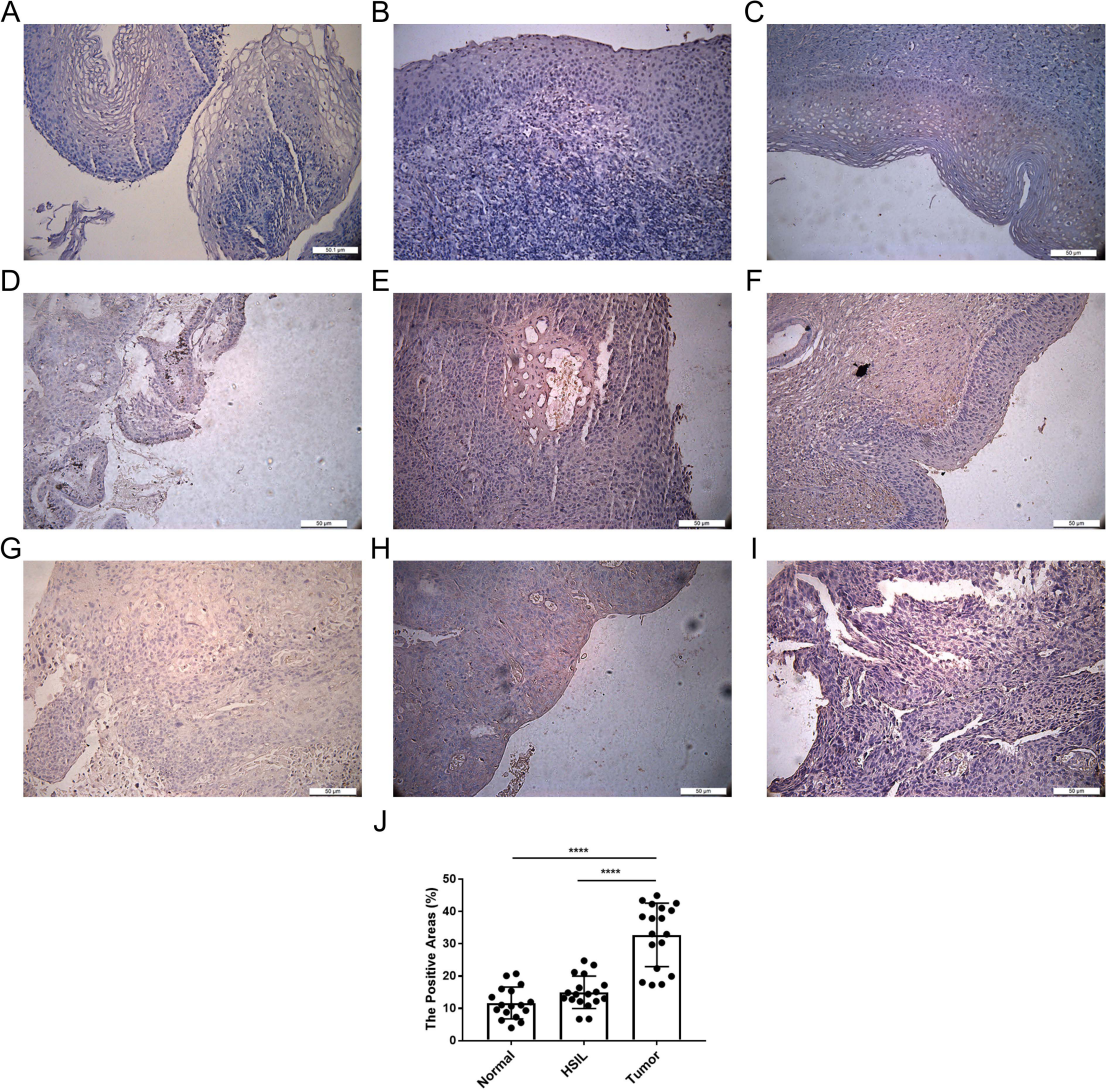
Expression of TFRC in cervical tissue. **(A-C)** IHC representative images of TFRC in normal cervical tissues. **(D-F)** images of TFRC in high-grade squamous intraepithelial lesions (HSIL). **(G-I)** IHC representative image of TFRC expression in cervical cancer tissue. **(J)** Positive area ratio of the IHC image shown. ****p < 0.0001.

The transferrin receptor TFRC is essential for the uptake of iron ions into cells. It plays a pivotal role in regulating cellular iron metabolism and maintaining iron balance. We investigated the protein expression of TFRC in 19 cervical cancer samples, 16 high-grade squamous intraepithelial lesions (HSILs), and 15 normal cervical tissues using immunohistochemistry (IHC) (**Figure 8**). We confirmed that TFRC expression was higher in cervical cancer tissues compared to normal cervical tissues (p < 0.0001) and HSIL (p < 0.0001) using IHC staining.

## 4 Discussion

Cervical cancer is the fourth most common type of cancers among women worldwide (21). While preventive strategies including HPV vaccination, routine screening protocols, and timely intervention for screen-detected preinvasive lesions have significantly reduced disease burden, the World Health Organization’s 2020 global elimination initiative sets ambitious 90-70-90 targets. This comprehensive strategy aims to reduce age-standardized incidence rates below 4 per 100,000 women annually through scaled-up prevention program (22). Nevertheless, the persistent clinical challenges in differentiating progressive cervical intraepithelial neoplasia (CIN) from transient HPV infections underscore the urgent need for robust molecular biomarkers enabling precise diagnosis and prognostic stratification. Our multi-cohort analysis systematically interrogated genomic datasets (GSE63514, GSE7803, GSE9750) complemented by TCGA cervical cancer profiles to identify survival-associated signatures. Through integrated bioinformatics pipeline analysis, we identified 20 hub genes (KIFC1, KIF14, HELLS, TK1, GINS2, WDR76, PCNA, DSG2, MCM5, SYNGR3, APOBEC3B, CHAF1B, TMPO, NUP62CL, RIBC2, PLA2G7, ARHGAP4, TFRC, GAD1, SPP1) demonstrating strongest correlation with overall survival (OS). Notably, transferrin receptor (TFRC) emerged as a prioritized candidate due to its dual role in cellular iron homeostasis and oncogenic signaling. However, the underlying mechanism of TFRC in the occurrence and malignant progression of cervical cancer is still unclear.

Transferrin receptor (TFRC) is a vital membrane protein that plays a critical role in cellular iron uptake, which is essential for various cellular processes, including growth and proliferation(Wenjing 19). Recent studies have shown that TFRC is closely associated with the development and progression of multiple cancer types. For instance, studies have shown that TFRC expression is significantly elevated in various cancer types, including breast, gastric, and colorectal cancers, where it correlates with poor prognosis and aggressive tumor behavior (23–25). Our preliminary findings revealing TFRC’s prognostic significance in cervical carcinogenesis warrant focused investigation into its pathobiological mechanisms and clinical translation potential.

Systematic pan-cancer analysis of unified TCGA datasets across 33 malignancies revealed distinct TFRC expression patterns. Our results showed that TFRC expression was higher in 12 tumors, including BLCA, BRCA, CESC, CHOL, COAD, ESCA, GBM, HNSC, LIHC, LUSC, STAD and UCEC (fold change >2.0, FDR<0.05). In contrast, it was expressed lower in KIRP, LUAD, PCPG, PRAD and THCA (p<0.01). Meanwhile, the expression of TFRC was correlated with pathological stage, lymph node metastasis, malignant degree of cervical lesions and HPV infection status. We confirmed the higher expression of TFRC in cervical cancer tissues than in normal cervix tissues, and its expression was higher in HSIL than in normal tissues using IHC staining. Higher expression was associated with reduced OS (p = 0.024), DSS (p = 0.009) and PFI (p = 0.007) in CESC. In different clinical stages, pathological T stages, and pathological N stages, higher TFRC expression was significantly associated with worse survival for OS and DSS. We constructed a nomogram model, TFRC contributed significantly to the prognosis and exhibited good predictive power for the OS and the DSS. Similarly, gastric cancer and prostate cancers models have shown that TFRC overexpression is associated with increased tumor incidence and aggressiveness, suggesting a direct link between iron uptake and cancer progression (26, 27). Sai Han et al. reported that TFRC were confirmed to be potential plasma diagnostic markers for Lymph node metastasis and lymphatic vasculature space infiltration in cervical cancer patients (28). Finally, the correlation between immune cell infiltration and TFRC expression was analyzed. We confirmed that immunosuppression in cervical cancer was closely related to high TFRC expression. The relationship between TFRC and immune cell function is a critical aspect of the tumor microenvironment (TME) that influences cancer progression and treatment outcomes. In the context of cancer, the expression of TFRC on immune cells can significantly affect their functionality and the overall immune response against tumors.

Understanding the mechanisms governing TFRC expression and function is crucial for developing therapeutic strategies targeting iron metabolism in cancer and other diseases. In cancer cells, due to the large amount of iron required for cell proliferation, the expression of TFRC often increases. Fu Wang et al. found that the absence of TFRC significantly impaired cell proliferation and migration *in vitro*, and significantly inhibited the growth and metastasis of HCC *in vivo*, while overexpression of TFRC had the opposite effect (29). Crawford Currie et al. reported that Colony growth suppression was often associated with the degree of simultaneous decrease in TFRC expression in prostate cancer (30). Beung-Chul Ahn et al. found that higher baseline TFRC levels predicted a favorable response to nivolumab in NSCLC with low PD-L1 expression (31). Moreover, the mechanisms underlying the regulation of TFRC expression in cancer cells are complex and involve various signaling pathways. For example, the transcription factor hypoxia-inducible factor (HIF) has been implicated in the upregulation of TFRC in response to low oxygen levels, a common feature in the tumor microenvironment. This adaptive response enables cancer cells to maintain iron homeostasis under hypoxic conditions, further promoting their survival (32). Additionally, mutations in oncogenes and tumor suppressor genes, such as TP53, have been shown to alter iron metabolism and TFRC expression, contributing to the malignant phenotype (33). Guofei Feng et al. reported that TfR was overexpressed in nasopharyngeal carcinoma, and TFRC knockdown inhibited nasopharyngeal carcinoma progression by suppressing the PI3K/Akt/mTOR signaling pathway (34). In studies of pancreatic and colon cancer cells, TFRC has been shown to modulate the MAPK signaling pathway, contributing to increased cell viability and resistance to apoptosis (35). Notably, the mechanistic basis of TFRC in cervical carcinogenesis and malignant progression remains unelucidated. To address this critical knowledge gap, we will undertake a pioneering study to comprehensively investigate its iron-dependent oncogenic mechanisms through integrated multi-omics approaches and functional validation models.

TFRC has emerged as a significant target in cancer therapy due to its overexpression in various malignancies, which is often associated with increased cellular iron uptake necessary for rapid tumor growth. The exploration of TFRC as a target has gained momentum in recent years, with numerous studies investigating its implications in both small molecule drug development and gene therapy applications aimed at enhancing therapeutic efficacy and specificity in cancer treatment. Some studies have demonstrated that transferrin-conjugated nanoparticles can effectively deliver chemotherapeutic agents directly to tumor cells, thereby minimizing systemic toxicity and enhancing therapeutic outcomes (25). Additionally, the use of small molecules that inhibit TFRC function has been explored as a potential strategy to disrupt iron homeostasis in cancer cells, thereby inducing ferroptosis, a form of regulated cell death characterized by iron-dependent oxidative stress (36). Moreover, the development of DNA aptamers that specifically bind to TFRC has opened new avenues for targeted therapy. These aptamers can serve as carriers for cytotoxic drugs, enhancing their selective uptake by cancer cells while reducing off-target effects (24). The high affinity and specificity of these aptamers for TFRC make them ideal candidates for the development of novel cancer therapeutics that could improve patient outcomes. Furthermore, studies have indicated that the combination of TFRC-targeting agents with existing chemotherapy regimens can enhance the overall efficacy of treatment, particularly in resistant cancer types such as breast and prostate cancer (27, 37). Recent advancements in nanoparticle technology have facilitated the development of TFRC-targeted gene delivery systems, which can effectively transport therapeutic nucleic acids to cancer cells. These systems can be engineered to enhance cellular uptake through receptor-mediated endocytosis, leveraging the overexpression of TFRC in tumor cells (38). Moreover, the use of CRISPR/Cas9 technology to edit TFRC expression levels in cancer cells has shown potential in preclinical models, suggesting that manipulating TFRC could alter tumor growth dynamics and response to therapy (39). In conclusion, the potential of TFRC as a target for both small molecule drugs and gene therapy represents a significant advancement in cancer treatment. The ongoing research into TFRC-targeted therapies could lead to the development of more effective and personalized treatment options for patients, particularly those with iron-dependent malignancies. As our understanding of TFRC’s role in cancer biology continues to evolve, it is likely that innovative therapeutic strategies will emerge, offering new hope for improved patient outcomes.

While this investigation provides novel insights into TFRC’s oncogenic role, three key limitations merit consideration: The current mechanistic understanding remains incomplete due to predominant reliance on bioinformatics analyses and preliminary experimental validation, necessitating integrated multi-omics approaches to fully delineate TFRC’s iron-mediated molecular circuitry. Furthermore, the restricted sample size in our single-center retrospective cohort introduces potential selection bias, limiting extrapolation to broader populations. Most critically, the therapeutic potential of TFRC-targeted strategies remains biologically unsubstantiated in the absence of preclinical models and early-phase clinical trials. To address these gaps, we plan to expand validation through multi-institutional cohorts, mechanistically dissect TFRC’s tumorigenic pathways via CRISPR-Cas9 screening coupled with spatial transcriptomics, and evaluate therapeutic efficacy using TFRC-specific PROTAC degraders in patient-derived xenograft models. These concerted efforts aim to bridge fundamental discoveries to clinical translation, potentially establishing TFRC as both prognostic biomarker and therapeutic vulnerability in cervical oncology.

## 5 Conclusion

In summary, based on GSE63514, GSE7803, GSE9750 and TCGA data, this study found that TFRC was highly expressed in cervical cancer, which was closely related to the prognosis of tumor and OS, DSS, and PFI. We confirmed the higher expression of TFRC in cervical cancer than in normal cervix tissues and HSIL using IHC staining. Meanwhile, the expression of TFRC was correlated with pathological stage, lymph node metastasis, malignant degree of cervical lesions and HPV infection status. Besides, we constructed a nomogram model that TFRC contributed significantly to the prognosis and exhibited good predictive power for the OS and the DSS. Finally, We confirmed that immunosuppression in cervical cancer is closely related to high TFRC expression. Thus, TFRC has certain diagnostic and prognostic value in cervical cancer, and may become a prognostic marker of cervical cancer.

## Data Availability

The sequencing data used in this study can be downloaded from the TCGA and GEO databases for free. The raw data are available from the corresponding author on reasonable request.
