# Supplementary material for: The TFRC as a prognostic biomarker and potential therapeutic target in cervical cancer: a preliminary study
Source: Front Oncol. 2025 Apr 15;15:1523137. doi: 10.3389/fonc.2025.1523137 (PMC12037406; doi:10.3389/fonc.2025.1523137)
Supplement: Supplementary file 1 [file DataSheet1.docx]

**Supplementary materials**

# Supplementary Table

Supplementary Table 1. Abbreviations and proper name

| Abbreviations | Proper name |
| --- | --- |
| ACC | Adrenocortical Carcinoma |
| BLCA | Bladder Urothelial Carcinoma |
| BRCA | Breast Invasive Carcinoma |
| CESC | Cervical Squamous Cell Carcinoma and Endocervical Adenocarcinoma |
| CHOL | Cholangiocarcinoma |
| COAD | Colon Adenocarcinoma |
| CPS | Combined positive score |
| CR | complete response |
| DCs | Dendritic cells. |
| DEGs | Differentially expressed genes |
| DLBC | Lymphoid Neoplasm Diffuse Large B-cell Lymphoma |
| DSS | Disease-specific survival |
| ESCA | Esophageal Carcinoma |
| GBM | Glioblastoma Multiforme |
| GC | Gastric cancer |
| GEO database | Gene Expression Omnibus database |
| GO | Gene Ontology |
| GO-BP | Gene Ontology-Biological Processes |
| GO-CC | Gene Ontology-Cell Component |
| GO-MF | Gene Ontology-Molecular Function |
| GTEx | Genotype-Tissue Expression |
| HNSC | Head and Neck Squamous Cell Carcinoma |
| IHC | Immunohistochemistry |
| KEGG | Kyoto Encyclopedia of Genes and Genomes |
| KICH | Kidney Chromophobe |
| KIRC | Kidney Renal Clear Cell Carcinoma |
| KIRP | Kidney Renal Papillary Cell Carcinoma |
| LAML | Acute Myeloid Leukemia |
| LGG | Brain Lower Grade Glioma |
| LIHC | Liver Hepatocellular Carcinoma |
| LUAD | Lung Adenocarcinoma |
| LUSC | Lung Squamous Cell Carcinoma |
| MAPK | Mitogen activated kinase-like protein |
| MESO | Mesothelioma |
| OS | Overall survival |
| OV | Ovarian Serous Cystadenocarcinoma |
| PD | progressive disease |
| PR | partial response |
| PRAD | Prostate Adenocarcinoma |
| pSD | Pathological stable disease |
| QIF | Quantitative fluorescence |
| READ | Rectum Adenocarcinoma |
| RVT | Residual viable tumor |
| SARC | Sarcoma |
| SD | Stable disease |
| SKCM | Skin Cutaneous Melanoma |
| STAD | Stomach adenocarcinoma |
| TCGA | The Cancer Genome Atlas |
| TGCT | Testicular Germ Cell Tumors |
| THCA | Thyroid Carcinoma |
| THYM | Thymoma |
| TIME | Tumor immune microenvironment |
| TISIDB | An integrated repository portal for tumor-immune system interactions |
| TMA | Tissue microarray |
| TME | Tumor microenvironment |
| UCEC | Uterine Corpus Endometrial Carcinoma |
| UCS | Uterine Carcinosarcoma |
| UVM | Uveal Melanoma |
